# Mitochondrial Pseudogenes in the Nuclear Genomes of *Drosophila*

**DOI:** 10.1371/journal.pone.0032593

**Published:** 2012-03-07

**Authors:** Hubert H. Rogers, Sam Griffiths-Jones

**Affiliations:** Faculty of Life Sciences, University of Manchester, Manchester, United Kingdom; The Centre for Research and Technology, Hellas, Greece

## Abstract

Mitochondrial pseudogenes in nuclear chromosomes (numts) have been detected in the genomes of a diverse range of eukaryotic species. However, the numt content of different genomes and their properties is not uniform, and study of these differences provides insight into the mechanisms and dynamics of genome evolution in different organisms. In the genus *Drosophila*, numts have previously only been identified on a genome-wide scale in the *melanogaster* subgroup. The present study extends the identification to 11 species of the *Drosophila* genus. We identify a total of 302 numts and show that the numt complement is highly variable in *Drosophilids*, ranging from just 4 in *D. melanogaster* to 67 in *D. willistoni*, broadly correlating with genome size. Many numts have undergone large-scale rearrangements in the nucleus, including interruptions, inversions, deletions and duplications of sequence of variable size. Estimating the age of the numts in the nucleus by phylogenetic tree reconstruction reveals the vast majority of numts to be recent gains, 90% having arisen on terminal branches of the species tree. By identifying paralogs and counting duplications among the extant numts we estimate that 23% of extant numts arose through post-insertion duplications. We estimate genus average rates of insertion of 0.75 per million years, and a duplication rate of 0.010 duplications per numt per million years.

## Introduction

It has been recognised that the transfer of genes into the nuclear genome has had a role in shaping the now minimal complement of functional genes contained within the organellar genomes of eukaryotes. Transfer of functional mitochondrial genes into the nucleus is believed to have last occured before the last common ancestor of all animals [1]. However, continued transfer of mitochondrial genome sequence into the nuclear genome occurs in almost all eukaryotic organisms [2], resulting in pseudogenic sequence of mitochondrial origin (numts). Numt sequence originates from all parts of the mitochondrial genome [3], [4], and contains mitochondrial noncoding sequence, tRNA and rRNA genes, fragments of protein-coding genes, and complete protein-coding genes that are untranslatable due to differences between the nuclear and mitochondrial genetic codes [5], [6]. Cases of numt insertions remodelling protein-coding genes to give rise to novel exon sequences have also been reported in yeast, human and plants [7].

The numts in the human genome have been the subject of detailed analyses since publication of the draft genome sequence [6], [8], [9], [10]. The data reveal continuous numt insertion over the last 58 million years of primate evolution. However, there is lack of consensus over whether the majority of human numts represent independent mtDNA insertions or arose by duplication events in the nuclear genome [6], [8]. In the horse genome, which contains very few duplicate copies of genes [11], the extant numt complement does not appear to contain any duplicates [12]. Analyses of the structure of numts in human and horse have revealed that many numts contain structural changes that have occured post-insertion, including inversions, deletions and insertions [2], [12]. Some numts are very large or complete copies of the mitochondrial genome; the largest human numt for example covers 90% of the human mitochondrial genome [10].

Identification of duplicate copies of numts in the human genome has been used to estimate the rate of duplication of unconstrained sequence, showing that a nucleotide is as likely to be involved in a large-scale duplication event as a point mutation [8]. Rates of duplication of eukaryote genes have been estimated to be between 0.020 and 0.002 per gene per million years for most eukaryotes [13]. Duplicated sequence may eventually be eliminated by deletion, the rate of which also varies considerably among organisms [14]. Studies using pseudogenes indicate that deletion of small DNA fragments proceeds ∼60 times faster in *Drosophila* (pseudogene half-life 14.3 m.y.) than in mammals (pseudogene half-life ∼884 m.y.) [15], [16].

Study of pseudogenes and mobile genetic elements are important for our understanding of rates of neutral evolution, duplication and deletion [14]–[19]. Rates of duplication and deletion of functionless sequence, along with numt insertion rates, vary among different organisms. Since numts have no self-replicating or transposition mechanism of their own, their study provides insight into mechanisms of evolution affecting the genome as a whole. Furthermore, pseudogenes are common in mammals but rare in *Drosophila* [20]. The lack of pseudogenes in *Drosophila* makes study of numts particularly valuable as they are easily detectable examples of sequence having no functional restraint.

In *Drosophila,* genome-wide annotation of numts has been limited to *D. melanogaster,* where just a handful of numt sequences have been detected [8], [21], [22], and three other members of the *melanogaster* subgroup [4]. A small number of numt-containing loci have been the subject of more detailed analyses in the *D. melanogaster* subgroup [23], [24], and the *D. ananassae* species cluster [25]. Beyond the *Drosophila* genus, the few insect genomes analysed have shown surprising variety in their numt content; from zero detected in *Anopheles gambiae* to ∼1,500 in *Apis mellifera*, the highest numt density of any animal studied [22], [26]. We describe the complement of numts across the *Drosophila* genus by annotating numts in the 11 species with sequenced nuclear and mitochondrial genomes. By predicting the age of numts and identifying orthologs and paralogs, we use the rate of numt insertion and duplication to provide insight into the evolutionary dynamics of unconstrained DNA sequence in *Drosophila*.

## Methods

### Annotation of numts

Numts were annotated in the 11 *Drosophila* species by searching the nuclear genome of each species (FlyBase 2008-07 release) with its mitochondrial genome (EMBL IDs: U37541, AF200833, AF200832, X03240, BK006335, BK006336, BK006337, BK006338, BK006339, BK006340, BK006341) using WU-BLASTN 2.0MP [27], with the hspsepSmax and hspsepQmax parameters (defining the maximum separation on the subject and query sequence respectively of high-scoring pairs (HSPs) that are combined) set to 50 bases, and an *E* value threshold of 10^−6^. Due to the highly A+T rich nature of the *Drosophila* mitochondrial genomes [28], we used the low-complexity filter NSEG [29] with standard settings to mask sequence that otherwise causes many spurious hits. We have excluded from the annotation a complete *D. melanogaster* mitochondrial DNA sequence currently included in the assembly on the “U” scaffold, which is likely to be the real mitochondrial genome, rather than part of the nuclear genome.

It should be noted that the *Drosophila* mitochondrial genome assemblies differ in their states of completion. Only *D. melanogaster, D. simulans, D. sechellia and D. yakuba* include the control region, which spans coordinates ∼14,000 to ∼20,000 in *D. melanogaster*. This region is A+T rich, repetitive and divergent in length and sequence [30].

Numts originating from the same mitochondrial DNA insertion are often no longer colinear due to subsequent nuclear rearrangement. Hits to the same scaffold that overlap or are separated less than 25 kb were grouped for potential merging. Each resulting group of hits was analysed manually to check for consistency in the micro-synteny between nuclear fragments and their mitochondrial origin. Groups that passed this check were then annotated and treated as a single numt, with breaks in synteny being annotated as gross rearrangement events of the following types:

- Internal interrupting sequence: non-numt annotated sequence interrupting sections of a single merged numt.
- Deletion: the numt contains non-contiguous pieces of mitochondrial DNA in the same orientation: stretches of missing sequence are likely the result of deletion of internal fragments.
- Inversions: reversed order of sections of the same numt.
- Internal duplications: chunks of numt sequence repeated with respect to the mitochondrial DNA.

We scanned all interrupting sequence for known repeat sequences using RepeatMasker open-3.3.0 [31] with the RM-20110914 libraries [32]. For each numt, we also retrieved all flanking and interrupting sequence within 200 bases of the annotated boundaries and searched for known repeat sequences. 69 out of 302 numts were located on extremely short scaffolds with <100b of sequence flanking the numt, and were excluded from the search. For the remaining 233, we counted the instances where the flanking and interrupting sequences contained known transposable elements, and recorded the class of those elements found.

### Estimating the age of insertion of numts

In order to estimate the insertion rate of the numts, we used the phylogenetic tree reconstruction method employed in the dating of human numts [8]. Each numt was aligned with the 11 *Drosophila* mitochondrial genomes using MAFFT v6.847b (2011/01/12) [33]. The *A. gambiae* sequence, which diverged from *Drosophila* ∼470 million years ago, was used as an outgroup in all alignments. All columns with a gap or ‘N’ character in any sequence were removed from the alignment. Using ‘dnaml’ from the ‘phylip’ suite version 3.69 [34] with default settings (no rate variation among sites, transition/transversion ratio of 2.0), a set of trees representing all possible divergence points of the numt with respect to the genus' mitochondrial genomes were tested to determine which best fits the alignment data for each numt. The tree topology used was that of the 12 *Drosophila* consensus phylogeny [20]. A window of date of insertion for each numt was then calculated, using the branch lengths of the most likely insertion point (from the tree that best fits the alignment data) and those not significantly less probable. Numts whose insertion date window extends past the *Drosophila-Sophophora* split were excluded from rate calculations.

### Finding paralogs and orthologs

To identify paralogs, all pairs of numts that originate from overlapping regions of the mitochondrial genome were aligned with the mitochondrial sequences of the host species and *A. gambiae*. The tree of the mitochondrial sequences was fixed to match the species tree [20] and dnaml was used to test the likelihood of all possible locations of the numt sequences. If the tree with the numts clustered is significantly more likely than the others (P<0.05), the numts are annotated as paralogs.

To identify orthologs, each pair of numts from different species whose insertions were dated to the same internal branches of the genus tree were aligned against the mitochondrial genomes of both host species and *A. gambiae*. As above, the tree arrangements were tested with dnaml. If the tree that has the numts clustered together is significantly more likely than the others then the numts are annotated as orthologs.

We attempted to confirm paralog annotations from the phylogenetic method by searching for micro-synteny conservation of the numts' flanking and interrupting sequences. We retrieved sequences encompassing each numt, including any interrupting sequence, and 10 kb of flanking sequence on each side. All numt-annotated, low complexity and repeat sequences were masked using NSEG and RepeatMasker as above. Numts located on short scaffolds or surrounded by repeat sequence (resulting in <200 unmasked bases) were discarded. Each masked sequence was searched against all other members of the paralog set using BLAST with an E-value threshold of 10^−6^. BLAST matches between flanking or interrupting sequences were taken as evidence of micro-synteny and thus confirmation of the paralog relationship.

### Calculating insertion and duplication rates

Rates of numt “gain” were calculated for each branch, using the time window for the age of each numt (see Estimating the age of insertion of numts) resolved into separate rates for insertion and duplication. Thus ortholog sets represent single insertion events, and each paralog group counts only as a single duplication, since DNA regions that have already duplicated show increased propensity for further duplication [35].

For the purpose of calculating rates, we assume insertions that are dated to a range of branches have equal probability of having inserted at any point during this window. In order to obtain a genus average insertion rate, we only use branches that arose in the last 20 million years. This includes the terminal branches leading to *D. melanogaster*, *D. simulans*, *D. sechellia*, *D. yakuba*, *D. erecta*, *D. mojavensis* and *D. virilis*, and the internal branches leading to *D. simulans/D. sechellia*, *D. yakuba/D. erecta*, and the *melanogaster* subgroup.

Insertion rate was calculated as follows:
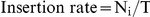
where N_i_ represents the number of insertions on chosen branches and T represents the total time along the these branches.

The duplication rate was calculated similarly using the same branches:
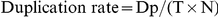
where Dp represents the number of duplications resulting in paralogs (only counting one event per paralog set), and N represents the average number of numts per genome. T represents half the total time elapsed since the start of the branches, as the mean age of duplications is assumed to be roughly half the length of the branch.

## Results

### Annotating numt sequences in *Drosophila* genomes

Numts were annotated in each of the 11 *Drosophila* genomes by searching the mitochondrial genome against the nuclear genome in each species. Overlapping and clustered hits from the same insertion were merged into single numt annotations. We annotated a total of 302 numts in the 11 *Drosophila* genomes (detailed in **Table S1**). The size of numt complement is highly variable in the genus (**Table 1**), ranging from just 4 in *D. melanogaster*, to 67 in *D. willistoni*. *D. virilis* has by far the largest total numt content, accounting for 147 kb of extant nuclear sequence. There is a positive correlation between genome assembly size and total numt content (in bps): Pearson *r* = 0.57 (P = 0.06), Spearman *r_s_* = 0.72 (P = 0.01). However there is no correlation between genome assembly quality measured by Q20 coverage [20] and numt content: Pearson *r* = 0.08 (P = 0.81), Spearman *r_s_* = 0.01 (P = 0.99).

**Table 1:** Numbers of numt annotations in 11 *Drosophila* species.

	Genome size*(% repeats)**	# numts annotated	Average length (bps)***	Total numt content (bps)
*D. melanogaster*	118 (5.35)	4	210	838
*D. simulans*	111 (2.73)	5	700	3,501
*D. sechellia*	115 (3.67)	25	1,502	37,553
*D. yakuba*	127 (12.04)	9	1,497	13,471
*D. erecta*	134 (6.97)	20	1,255	25,103
*D. ananassae*	176 (24.93)	26	1,537	39,952
*D. persimilis*	138 (8.47)	54	1,335	72,107
*D. willistoni*	187 (15.57)	67	900	60,284
*D. mojavensis*	172 (13.96)	24	3,029	72,689
*D. virilis*	161 (8.92)	59	2,506	147,862
*D. grimshawi*	138 (2.84)	9	2,075	18,673

* Genome size estimated by assembly size [20].

** Repeat content annotated by ReAS [48], excluding scaffolds <200 kb [20].

*** Average numt length excludes internal duplications and interrupting sequence between merged fragments.

Our *D. melanogaster* numt annotation is consistent with previous studies, which have reported six [21], five [22], and three [3] numts, though each study has used different search parameters and merging methods. Hazikani-Covo et al. [4] report many more numts in the *Drosophila* subgenus species they searched; the excess is likely due to low complexity sequences in the AT-rich *Drosophila* mitochondrial genome, as we identified similar numbers before removing AT-rich alignments.

**Figure 1:**
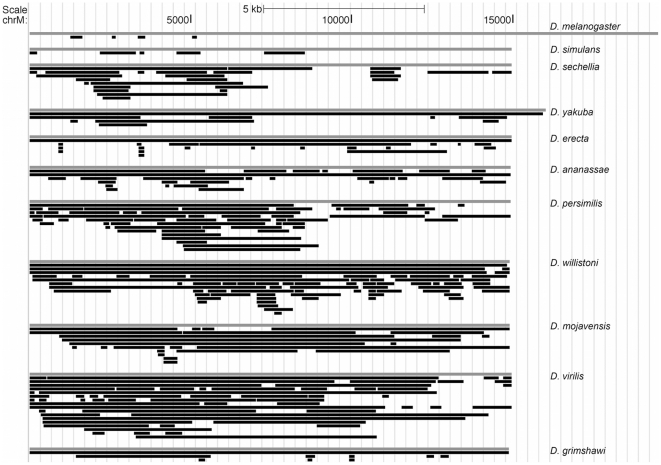
Mitochondrial origins of numts. Locations of origin of numt sequences (black) in the mitochondrial genomes (grey) for 11 *Drosophila* species are shown. The *D. melanogaster* mitochondrial assembly is relatively large, because it has a larger portion of the variable control region sequenced.

The numt content of the remaining *Drosophila* species has not previously been reported. The numts originate from all parts of the mitochondrial genome (**Figure 1**). On first inspection, the data suggest biases in numt origin. However, under-represented regions may have been masked A+T rich sequence, and other regions may be over-represented due to post-insertional duplication. Eleven numts span over half the length of the mitochondrial genomes, of which three (in *D. erecta*, *D. ananassae* and *D. grimshawi*) represent complete or almost complete mitochondrial genomes. There is no correlation between numt size and percentage identity with the mitochondrial sequence (Pearson *r* = −0.03; P = 0.51).

The average length of numt sequence across the genus is 1.5 kbps—approximately 8% of a *D. melanogaster* mitochondrial genome. There is however considerable variation in species-specific average numt length, from just 210 bases in *D. melanogaster* to over 3 kb in *D. mojavensis* (95% CI: 965–2043 bp). *D. willistoni* is unusual in being the only species with a large number of numts that are well below average size—67 numts with an average length of 900 bases.

By manual inspection of numt-mitochondrial DNA alignments, we find evidence of large-scale post-insertional rearrangements in the larger numts of each *Drosophila* species. We find that a total of 79 (26%) annotated numts have undergone gross rearrangements: instances of interrupting sequence, deletions, inversions and internal duplications (**Table 2**). The amount of sequence affected by the local rearrangement events is highly variable, ranging from the minimum detectable size of 200 bases (see Methods) to ∼25 kb. The average sizes of deletion and duplication are similar at ∼2 kb, with standard deviations of 2.9 kb and 2.0 kb respectively, while the average insertion size is larger at 5.5 kb with a high degree of variation (standard deviation = 7.9 kb).

**Table 2:** Number of gross rearrangement events for each *Drosophila* species.

	Interruptions	Deletions	Inversions	Duplications
*D. melanogaster*	-	-	-	-
*D. simulans*	-	-	-	-
*D. sechellia*	2 (2)	1 (1)	1 (1)	-
*D. yakuba*	4 (2)	5 (3)	-	1 (1)
*D. erecta*	-	4 (2)	-	-
*D. ananassae*	5 (5)	-	-	-
*D. persimilis*	-	5 (4)	2 (2)	1 (1)
*D. willistoni*	21 (8)	12 (7)	1 (1)	4 (4)
*D. mojavensis*	12 (6)	4 (3)	-	3 (3)
*D. virilis*	15 (8)	16 (11)	2 (2)	9 (5)
*D. grimshawi*	-	1 (1)	-	-
**Total**	**59 (31)**	**48 (32)**	**6 (6)**	**18 (14)**
**Average size/kb [s.d.]**	**5.7 [8.0]**	**2.0 [2.9]**	**0.55 [0.32]**	**1.8 [2.0]**

Interrupting sequence, deletions, inversions and duplications affecting at least 200 bases of numt sequence in the nuclear genomes after insertion are shown. The number of distinct numts affected is shown in parentheses. Multiple rearrangements of the same type were only counted once for each numt.

The interruptions to numts may either have arisen due to insertion of mobile genetic elements, or they could be the result of large-scale inversions involving a numt and its flanking region. We scanned all interrupting sequences within the numts for known *Drosophila* repeat sequences. 45% of the total interrupting sequence from 59 interruptions is composed of interspersed repeat sequence. In the genus as a whole, the vast majority of interrupting repeat sequence derives from LTR and LINE-like retrotransposons—116 kb in 31 interruptions compared with 17 kb of DNA transposon sequence in 24 interruptions. Of the remaining 55% of interrupting sequence that is not composed of known mobile genetic elements, only 1.5% is composed of simple repeats and low complexity sequence. Four interruptions in *D. willistoni* numts and two in *D. mojavensis* numts contain exons of protein-coding genes.

In order to assess whether numts tend to colocalise with repeat sequences, we searched the flanking and interrupting sequences of all numts for repetitive elements located within 200 bases of the numt. Excluding 69 numts for which we could not retrieve sufficient flanking sequence, 92 of 233 (39%) were found to be adjacent to at least one annotated repeat. In contrast, an average of only 15% of genus non-protein coding DNA is composed of repeat sequence [20], suggesting a significant association between *Drosophila* numts and repetitive elements (Fisher's exact test; P<0.01).

In total, 24 numts (8%) are located in the introns of annotated protein coding genes. FlyBase (2011-05 release) annotates 21 Gln^(TTG)^ tRNAs and one Tyr^(GTA)^ tRNA within the boundaries of our annotated numts. None of these are located in the interrupting sequences discussed above, rather they derive from mitochondrial tRNAs. Scanning these sequences with tRNAscan-SE 1.23 [36] on maximum sensitivity (covariance model only) reveals that the Gln^(TTG)^ tRNAs are detected only with organellar tRNA-specific covariance models, while the Tyr^(GTA)^ tRNA present in *D. willistoni* scores highly with both organellar and nuclear models.

### Orthologs and paralogs

**Figure 1** shows that some numts appear to derive from identical or nearly identical regions of the mitochondrial genome. In our analysis, numt “gains” include two types of event: original mtDNA insertions and subsequent duplications of numt sequence. Thus numts overlapping in their mitochondrial region of origin may either have arisen by chance repeated insertion of the same fragment of mtDNA, or by duplication of an original numt in the nucleus. We distinguish between these by constructing an alignment of the two overlapping numts and the host species' mitochondrial genome, and testing all possible tree topologies. Similarly we test for orthology of numts from different genomes by aligning numts dated to the same internal branches of the genus tree with their host species' mitochondrial genomes, and testing all tree configurations.

**Figure 2:**
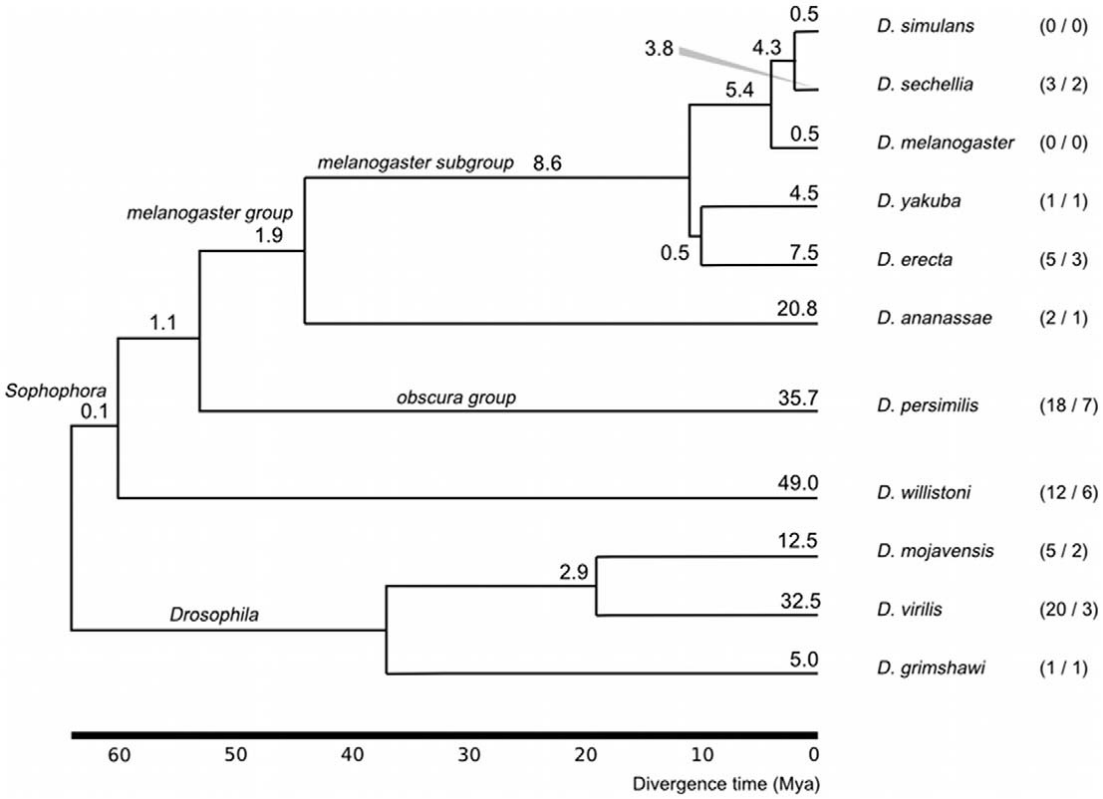
Age of numt insertions. Average frequencies (insertions per million years) of numt insertions on each branch of the *Drosophila* tree are shown. In parentheses is the number of extant numts that have arisen by duplication (left), and the number of distinct paralog sets (right). Divergence times were derived from TimeTree [47] and the tree toplogy from [20].

We find 93 *Drosophila* numts that have at least one paralogous copy (35% of total). These comprise 26 groups of paralogs, each group originating from a single numt (see **Figure 2** and **Table S2**). Therefore 67 numts (25%) have arisen through duplication from preexisting mtDNA insertions. *D. persimilis*, *D. willistoni* and *D. virilis* contain the most duplicated numts.

We attempted to confirm the paralog groups predicted by phylogenetics using micro-synteny conservation. To this end, we searched for sequence similarity in the flanking and interrupting sequences of all paralog assignments. 9 of the 26 paralog sets could not be tested due to the location of member numts on short scaffolds or surrounded by masked repeat sequence – 6 of these share at least one boundary of annotation with identical mitochondrial genome coordinates. Members of 14 paralog sets showed clear sequence similarity between flanking and/or interrupting sequence. The remaining 3 sets consisted of only 2 numts each, with the mitochondrial origin of one numt entirely encompassed by the other, consistent with partial duplication of an internal section of a numt.

We find fewer orthologous relationships: only 15 *Drosophila* numts from nine paralog sets are predicted to have orthologs in the genus, all being from species within the *melanogaster* group. These orthologous relationships form four distinct ortholog sets (see **Table S3**). The largest is conserved in *D. melanogaster*, *D. simulans* and *D. sechellia*, and is therefore predicted to have arisen in the common ancestor of the *melanogaster* subgroup at least four million years ago. The other sets are conserved in only two species each: *D. simulans* and *D. sechellia*, *D. yakuba* and *D. erecta*, and the oldest *D. sechellia and D. yakuba*. The last is predicted to have arisen in the common ancestor of the *melanogaster* subgroup at least 11 million years ago.

### Estimating the age of insertion of numts

The synonymous substitution rate of *Drosophila* nuclear genes is 4.5–9.0 times lower than that of mitochondrial genes [37]. Thus numts are subject to slow substitution rate in the nucleus, and we are able to date them using sequence similarity to a range of closely related mitochondrial genomes, as previously reported [8]. By comparing each numt against the alignment of the 11 *Drosophila* mitochondrial genomes, and choosing the best tree from a set representing each possible divergence point, we have estimations of the age of each numt in the nuclear genome. **Figure 2** shows the frequency of insertions on each branch of the *Drosophila* phylogeny. Since for some numts there are multiple branches not less significantly probable than the best branch, we use a measure of frequency ‘density’ that distributes the frequency across the potential insertion branches in proportion to each branch's length.

The vast majority of *Drosophila* numt insertions (238.7, 89.7%) are assigned to terminal branches, and only 27.3 (10.3%) are predicted to have arisen in the last common ancestor of the 11 species. The age windows of 36 numts include the *Drosophila—Sophophora* divergence at the base of the tree. However, many of these have large uncertainty in the age-range due to poor alignments, and were hence excluded from further analyses. It is also possible that among these are recombinant sequences, derived from multiple pre-existing numts of varying age—such recombinant numts are known to be common in some taxa [38].

### Rates of insertion, duplication and deletion

The average rate of numt gain, measured using the number of numts predicted to have arisen on only the branches of the tree up to ∼18 million years old, is 1.26 per million years. Excluding numts that have arisen by duplication, we calculate an average rate of numt insertion in *Drosophila* of 0.95 per million years. However rates in the genus vary considerably (see Table 3). The fastest rates are observed in *D. sechellia* (1.92 per million years) and *D. virilis* (1.71 per million years), while the slowest rates of insertion are found in *D. melanogaster* (0.12 per million years), *D. simulans* (0.23 per million years), and *D. grimshawi* (0.14 per million years). The remaining six species have insertion rates between 0.45 and 0.80 per million years. The calculated rates of insertion do not correlate with genome size (Pearson *r* = 0.11; P = 0.75).

**Table 3:** Insertion and duplication rates for each lineage.

Branch	Insertion rate (insertions per m.y.)	Duplication rate – one per set(duplications per numt per m.y.)	Duplication rate – total(duplications per numt per m.y.
*D. melanogaster*	0.12	-	-
*D. simulans*	0.23	-	-
*D. sechellia*	1.92	0.063	0.094
*D. yakuba*	0.45	0.006	0.006
*D. erecta*	0.75	0.039	0.064
*D. ananassae*	0.47	0.002	0.004
*D. persimilis*	0.66	0.005	0.012
*D. willistoni*	0.80	0.003	0.006
*D. mojavensis*	0.66	0.011	0.028
*D. virilis*	1.71	0.006	0.041
*D. grimshawi*	0.14	0.009	0.009

Duplication rates were calculated both including and excluding multiple duplications from the same paralog set, and using only terminal branches of the tree. No duplications were observed in *D. melanogaster* and *D. simulans*.

To calculate a genus average duplication rate we used the same recent branches, and counted each paralog set as only one duplication, since DNA regions that have already duplicated have a higher propensity for further duplication [35]. We thus estimate an average duplication rate of 0.010 per numt per million years. Since there are so few numts dated to internal branches, it is difficult to directly calculate a numt deletion rate from inferred losses. However, considering simply the rate of numt gain and assuming a steady state, we estimate a deletion rate of 0.052 deletions per numt per million years.

## Discussion

Previous annotation of numts in *Drosophila* genomes is limited to *D. melanogaster* [8], [21], [22], and three other members of the *melanogaster* subgroup [4]. The present study has identified numts in 11 species of the *Drosophila* genus with both nuclear and mitochondrial genome sequenced. While it is important to note that numt annotations are prone to biases from the varying quality of assembly of the *Drosophila* genomes [20], we find that the numbers of numts is highly variable—from 4 in *D. melanogaster* to 67 in *D. willistoni—*and are not correlated with genome assembly coverage. It is noteworthy that *D. melanogaster* has the smallest numt complement and should not be considered representative of the genus.

A correlation between genome size and total numt content in base pairs has been previously proposed [3], [4], [39] and while the variation in *Drosophila* genome sizes is relatively small, we do find a correlation with total numt sequence. Larger genomes may be more susceptible to foreign DNA integration due to more frequent spontaneous double strand breaks, and larger genomes tend to lose less DNA [4]. Yet intriguingly, we do not find a correlation between insertion rates and genome size. Our rates of numt insertion for individual species are based in some lineages on few insertions and in others on long terminal branches, and are therefore subject to significant error. However, it is clear that considerable variation does exist. The two species with the highest estimated insertion rates, *D. sechellia* and *D. virilis*, have among the smallest and largest genome assemblies respectively, and each has a much larger numt content than its closest related species (*D. simulans* and *D. mojavensis* respectively). Thus we speculate that the lack of correlation between genome size and numt insertion rates may reflect variation in the rate and size-spectrum of DNA loss between species [21].

There is also large variation in average length of numts between species, with the *D. willistoni* numts being strikingly small. Given the frequency of post-insertional gross mutations, this may reflect the frequency and size-spectrum of subsequent deletions, rather than the size of the original insertions. We suggest that the particularly fragmented numt complement of *D. willistoni* may be connected with this species' high transposon activity and enlarged genome [20]. However, *D. ananassae* has a similarly large genome with high repeat activity, and its numts are not particularly short. *D. ananassae* is unusual in that it has a numt complement smaller than expected given the correlation with genome size, and many of its numts appear to have arisen by duplication.

On average, 63% of the *Drosophila* genome assemblies represent non-protein coding DNA, and 24% of this non-protein coding fraction is intronic [20]. Although the most recent human numts have tended to insert in introns [40], the low incidence of *Drosophila* numts in introns (8%) is similar to that in the honeybee [26]. *Drosophila* introns are highly conserved [20], and numt integration may perturb transcriptional regulation by altering intron length or disrupting sequence signals that regulate gene expression [41].

Our analysis of large-scale rearrangements in numts shows that insertion, deletion and rearrangement events subsequent to integration are common in *Drosophila*. Despite the fast turnover, 26% of numts have been affected by at least one gross mutation, with many of the larger numts having been affected by several. Approximately half of the interrupting sequence is composed of known repeat sequences, mainly LTR and LINE-like retrotransposons, which are the most abundant transposable elements in the genus [20]. These interruptions may be the result of direct insertion of mobile genetic elements into numts, or large inversions that result in numt sequence being interspersed with the neighbouring sequence. Six cases where segments of numts surround exons of protein-coding genes are likely to be among the latter. However, we find that the association of numts and repeats is also evident at very close distances, which is less likely to be due to rearrangements. 39% of numts are located within 200 bases of repetitive elements, significantly more than expected given the average repeat content of *Drosophila* non-protein coding DNA is only 15%. It has been suggested that the location of transposable elements may influence the integration of numts in humans [42], and our data is consistent with the same phenomenon in *Drosophila*.

We find that the vast majority of *Drosophila* numts have recent origins: 90% of extant numts are predicted to have been inserted on terminal branches of the genus tree. This observation raises the concern that old divergent numts may be missing from our annotation due to accumulation of mutations in both nuclear and mitochondrial sequences. If our sequence similarity search has limited numt detection, we might expect to have biased against shorter numts, which are less likely to meet a blanket BLAST threshold. This would lead to higher identity on average for shorter numt annotations. Since we do not observe any correlation between numt length and divergence, we suggest that numt transfer is an ongoing process with a high rate of turnover.

By identifying paralogous relationships among the numts of each species, and thus distinguishing between gain events by duplication and by independent transfer, we have estimated both a rate of insertion and a rate of duplication for the numts of *Drosophila*. The average rate of numt insertion we find in *Drosophila* is 0.95 per million years, which is lower than the rate calculated for human of 5.1 per million years [8]. We hypothesise that the lower rate is due to the smaller and more compact *Drosophila* genomes, offering less opportunity for non-deleterious integrations. Although we only use short and recent branches for the genus average rate calculations, it is known that some DNA integrants have very short life spans [43], and even some recently inserted numts may have been subsequently deleted. Thus the rates we calculate should be considered lower-bound estimates.

In line with our prediction of the young age of most numts, we detected remarkably few orthologs among the numts of *Drosophila* using the phylogenetic tree reconstruction method. Only 17 numts are predicted to have extant orthologs in other species, forming 9 distinct sets all belonging to the *melanogaster* group. The absence of predicted orthologs among the larger numt complements of the remaining species (*D. ananassae*, *D. persimilis*, *D. willistoni* and the *Sophophora* subgenus) is not necessarily surprising considering the longer divergence times between even the closest of those species. Our identification of duplications is limited by the continued presence of each copy in the genome, and by our strict criteria for ortholog and paralog classification by the tree testing method. Thus we have likely underestimated the number of numts that have arisen by duplication.

We estimate a numt duplication rate in the *Drosophila* genus of 0.010 duplications per numt per million years. This is an order of magnitude higher than the rate reported in human, estimated to be 0.0022 duplications per numt per million years [8]. Bensasson and colleagues noted that the duplication rate they calculated for numts in humans was of the same order as the rate of point substitution for noncoding DNA (0.0016–0.0025 per site per million years) [14], [44]. Invertebrates have a faster synonymous substitution rate of 0.016 mutations per site per million years [14]. Our calculations therefore suggest that a site is also approximately as likely to be duplicated as substituted in *Drosophila*. Assuming a constant frequency of numts and a steady decay curve, we find a half-life of numts in *Drosophila* of 18 million years and a deletion rate of of 0.052 per numt per million years. This estimation broadly fits with pseudogene studies that indicate a high rate of DNA loss in *Drosophila*, with a pseudogene half-life of 14.3 million years [15], [45].

The turnover of functional genes in *Drosophila* has been estimated at 0.0012 gains and losses per gene per million years for the the entire complement of genes [46]. By a different method the rate of duplication of genes in *Drosophila* has been estimated at 0.0023 duplications per gene per million years [13]. Thus we estimate a rate of duplication of unconstrained sequence which is roughly an order of magnitude faster than the duplication rate observed for protein-coding sequence, while fitting in the general range found among eukaryotes of ∼0.002 to 0.020 per gene per million years [13].

In conclusion, we have annotated numts in 11 species of the *Drosophila* genus; a detailed analysis of the numts of the genus has not previously been attempted. We highlight unexpected variations in numt complement between species, broadly correlated with genome size. Numt insertions are generally young, and yet many show evidence of large-scale post-insertion rearrangement events. Our rate calculations for numt insertion and mutation provide useful estimates of typical gain and loss dynamics of passive non-functional sequence in the *Drosophila* genus.

## Supporting Information

Table S1 **List of numts with the coordinates of their nuclear chromosomal locations and their mitochondrial genome origin.** Numts that have been merged from multiple separate BLAST hits are represented by the details of individual hits.
(XLS)

Table S2 **List of paralogous numts within each species.** Each row represents a paralog set.
(XLS)

Table S3 **List of orthologous numts between species.** Each row represents an ortholog set, and each cell is an individual numt or a group of paralogous numts.
(XLS)
